# Editorial: Cancer prevention and therapy using herbal formulations of natural immune modulators

**DOI:** 10.3389/fimmu.2026.1815940

**Published:** 2026-03-11

**Authors:** Vinay Kumar, Mohamad Taleuzzaman

**Affiliations:** 1 Department of Pharmaceutical Chemistry, KIET Group of Institutions (KIET School of Pharmacy), Ghaziabad, India; 2 Department of Pharmaceutical Chemistry, Faculty of Pharmacy, Maulana Azad University, Jodhpur, India

**Keywords:** ashwagandha, curcumin, immune modulators, *Tinospora cordifolia*, Traditional Chinese Medicine (TCM)

Cancer continues to be a global health challenge, with conventional therapies often limited by toxicity, resistance, and reduced patient quality of life. Herbal formulations containing natural immune modulators are emerging as promising adjuncts in both prevention and therapy. These plant-derived compounds can enhance immune surveillance, regulate inflammatory pathways, and potentially reduce cancer risk while supporting conventional treatments (1, 2).

## Mechanisms of action

Herbal immune modulators exert their effects through diverse biological pathways:

- Immune Enhancement Stimulate T-cell and NK-cell activity, increase cytokine production (IL-2, IFN-γ).
- Anti-Proliferative Effects Inhibit tumor cell growth and angiogenesis.
- Apoptosis Induction Trigger programmed cell death in malignant cells.
- Reversal of Drug Resistance Modulate efflux pumps and restore sensitivity to chemotherapy.
- Regulation of Autophagy & Ferroptosis Influence cell survival mechanisms linked to cancer progression.

## Examples of herbal immune modulators

**Table 1:** Examples of immune modulators.

Herb/Compound	Key Properties	Potential Role in Cancer
Curcumin (Turmeric)	Anti-inflammatory, antioxidant	Enhances immune response, inhibits tumor growth
*Withania somnifera* (Ashwagandha)	Adaptogen, immunomodulator	Supports apoptosis in cancer cells, improves resilience
Green Tea Polyphenols (EGCG)	Antioxidant, anti-proliferative	Suppresses angiogenesis, boosts immune defense
*Ganoderma lucidum* (Reishi mushroom)	Immunostimulant	Enhances NK-cell activity, improves quality of life
Tinospora cordifolia (Guduchi)	Immunomodulatory	Strengthens immune system, reduces chemotherapy side effects

## Benefits and challenges

**Benefits:**

- Lower toxicity compared to synthetic drugs
- Synergistic effects with conventional therapies
- Improved patient quality of life and immune resilience

**Challenges:**

- Variability in herbal composition and dosage
- Limited large-scale clinical trials
- Risk of herb-drug interactions
- Need for standardized formulations and regulatory oversight

Tumor microenvironment (TME) remodeling and immune re-education are replacing direct cytotoxicity as the primary methods of cancer prevention and treatment. Using systems immunology, multi-omics, and molecular pharmacology, this Research Topic highlights the translational potential of herbal formulations rich in natural immune modulators, especially from Traditional Chinese Medicine (TCM) and Indian ethnomedicine Liang et al.

The relationship among tumor biology, immunological surveillance, inflammation, and psychological stress is a major issue. According to a study on Xiaoyao San (XYS) in triple-negative breast cancer (TNBC), persistent emotional distress causes neuroendocrine-immune dysregulation, which speeds up the growth of tumors. High-dose XYS activated the intratumoral Rela/NF-κB–Cxcl9 axis, which improved CD8^+^ T-cell infiltration and cytotoxicity, and reduced tumor growth and depressive-like behavior in a CUMS model. Network pharmacology, transcriptomics, and molecular dynamics are used in multi-layered validation to highlight identified molecular nodes (Rela/p65) as immunoregulatory targets and enhance mechanistic credibility Liu et al.

In addition, resistance and immune-related adverse events (irAEs) in solid tumors are covered in the evaluation of TCM-assisted immune checkpoint inhibitor (ICI) therapy. By increasing T-cell activation, decreasing myeloid-derived suppressor cells (MDSCs), modifying cytokine networks, and minimizing inflammatory toxicities, TCM formulations seem to improve ICI efficacy. Though there are still issues with standardization, pharmacokinetics, herb-drug interactions, and regulatory harmonization, the multi-target, low-toxicity paradigm conceptually fits with systems immunology Cui et al.

TCM-derived chemicals as adjuvants for tumor vaccines represent another frontier. These substances fall into the following categories: non-canonical immune regulators, delivery enhancers, immunostimulatory modulators, and integrated platforms. These substances serve as immune-educating agents that enhance dendritic cell maturation, antigen presentation, and T-cell priming instead of directly causing cytotoxicity. This strategy is especially pertinent for customized neoantigen vaccines. Thorough clinical validation is still necessary, though Nair et al.

The immune microenvironment modification is further supported by the preclinical assessment of Yiai Fuzheng decoction (YFD) in TNBC. YFD decreased MDSCs and tumor-associated macrophages, changed macrophage polarization toward an M1 phenotype, and enhanced CD4 and CD8 T-cell infiltration. The multi-level activity of herbal immunomodulators was demonstrated by integrated metabolomics and transcriptomics, which showed the Warburg effect, MEK/ERK signaling, and reprogramming of arginine metabolism. 5-year follow-up research combining iodine-125 seed brachytherapy and Chinese herbal medicine (CHM) in advanced non-small cell lung cancer (NSCLC) demonstrates clinical value. Pending randomized validation, comparable survival outcomes to chemotherapy plus brachytherapy, combined with fewer problems, point to better tolerance and perhaps quality-adjusted survival benefits Yu et al.

In an AOM/DSS colon cancer model, processed triterpenoids from Sanguisorba officinalis also offer mechanistic insights. Through NF-κB inhibition and TNF-α lowering, processed forms demonstrated greater preventative efficacy. Using molecular evidence, the integration of UPLC-Q-TOF-MS, network pharmacology, molecular docking, and DNA methylation analysis rationalizes conventional processing Li et al.

Beyond TCM, ethnomedicinal herbs from Tamil Nadu, such as Curcuma longa and Withania somnifera, show promise against cancer by promoting apoptosis, inhibiting angiogenesis, modifying the immune system, and promoting therapeutic synergy. However, there are still significant obstacles to translation, including low bioavailability, toxicity issues, a lack of standardization, and a dearth of clinical trials Gan et al.

Collectively, the articles in this Research Topic converge on several unifying principles:

1. Immune modulation is central to the anticancer effects of herbal formulations.
2. Multi-omics and systems biology tools are essential for mechanistic elucidation.
3. Combination strategies—with ICIs, radiotherapy, or vaccine platforms—represent the most promising translational pathway.
4. Standardization, safety evaluation, and high-quality clinical trials are critical for global acceptance.

As guest editors, we believe this compilation reflects a critical inflection point in herbal oncology research. The field is transitioning from descriptive ethnopharmacology to mechanism-based, immune-centered precision therapeutics. Natural immune modulators are not substitutes for modern oncology but may serve as synergistic agents that enhance efficacy, reduce toxicity, and restore immune competence.

Future research should prioritize biomarker-driven clinical trials, pharmacokinetic profiling of multi-component formulations, AI-assisted network modeling, and international regulatory harmonization. Interdisciplinary collaboration among oncologists, immunologists, pharmacologists, and traditional medicine experts will be essential to translate these promising findings into evidence-based integrative cancer care.

In conclusion, herbal formulations rich in natural immune modulators represent a fertile intersection of tradition and innovation. By illuminating molecular targets such as NF-κB, TNF-α, and immune cell infiltration pathways, the studies in this Research Topic collectively advance the scientific foundation for integrative cancer prevention and therapy. The challenge ahead lies not in proving that herbs work, but in defining how, for whom, and in what combination they can most effectively contribute to the global fight against cancer.

## Conclusion

Herbal formulations containing natural immune modulators represent a promising frontier in cancer prevention and therapy. Evidence supports their role in enhancing immunity, reducing side effects, and complementing conventional treatments. However, rigorous clinical validation, standardization, and regulatory frameworks are essential before widespread adoption. Integrating traditional wisdom with modern biomedical research could pave the way for safer, more effective cancer management strategies.
